# *In silico* study of the effects of anti-arrhythmic drug treatment on sinoatrial node function for patients with atrial fibrillation

**DOI:** 10.1038/s41598-019-57246-5

**Published:** 2020-01-15

**Authors:** Jieyun Bai, Yaosheng Lu, Henggui Zhang

**Affiliations:** 1 Department of Electronic Engineering, College of Information Science and Technology, Jinan University, Guangzhou, China; 2 Biological Physics Group, School of Physics & Astronomy, The University of Manchester, Manchester, United Kingdom

**Keywords:** Atrial fibrillation, Atrial fibrillation

## Abstract

Sinus node dysfunction (SND) is often associated with atrial fibrillation (AF). Amiodarone is the most frequently used agent for maintaining sinus rhythm in patients with AF, but it impairs the sinoatrial node (SAN) function in one-third of AF patients. This study aims to gain mechanistic insights into the effects of the antiarrhythmic agents in the setting of AF-induced SND. We have adapted a human SAN model to characterize the SND conditions by incorporating experimental data on AF-induced electrical remodelling, and then integrated actions of drugs into the modified model to assess their efficacy. Reductions in pacing rate upon the implementation of AF-induced electrical remodelling associated with SND agreed with the clinical observations. And the simulated results showed the reduced funny current (*I*_*f*_) in these remodelled targets mainly contributed to the heart rate reduction. Computational drug treatment simulations predicted a further reduction in heart rate during amiodarone administration, indicating that the reduction was the result of actions of amiodarone on *I*_*Na*_*, I*_*Kur*_*, I*_*CaL*_*, I*_*CaT*_*, I*_*f*_ and beta-adrenergic receptors. However, the heart rate was increased in the presence of disopyramide. We concluded that disopyramide may be a desirable choice in reversing the AF-induced SND phenotype.

## Introduction

Sinus node dysfunction (SND) is associated with abnormal sinoatrial node (SAN) impulse formation resulting in sinus bradycardia. As the elderly population continues to increase, SND is becoming an increasingly common medical condition in patients with atrial fibrillation (AF)^1^. AF and SND often coexist and interact in clinical practice, but the causal link between these two arrhythmias remains unclear^2,3^. AF itself may alter the function of the normal SAN or promote pre-existing SND. AF is believed to shut down the normal function of the SAN by long-term overdrive suppression of its activity^4^. AF-induced SND was evidenced by slowed intrinsic heart rate, which was gradually reversed after the termination of AF^5–7^. The intrinsic heart rate was jointly regulated by the voltage (cyclic activation and deactivation of membrane ion channels) and calcium clocks (rhythmic spontaneous sarcoplasmic reticulum calcium release)^8^. In the pacing-induced canine model of AF, changes in membrane ion channels and calcium handling proteins imply the impact of the voltage and calcium clocks. Alterations in hyperpolarization-activated cyclic nucleotide-gated channels HCN4, slow delayed-rectifier α-subunit KvLQT1, L-type/T-type calcium current subunit Cav1.2/Cav3.1^6^ and calcium handling proteins^9^ were observed in SAN cells, suggesting that AF may lead to SAN remodeling and thereby SND.

It was shown in large-scale clinical studies that amiodarone is the most effective drug for maintaining sinus rhythm in patients with AF^10–13^. However, amiodarone impairs SAN function in one-third of AF patients^14^. Touboul *et al*. showed that amiodarone has no effects on sinus cycle length (CL) in pacing-induced AF patients^15^, whereas Hoffmann *et al*. showed that it depresses SAN function in some patients with sick sinus syndrome^16^, suggesting that amiodarone cannot be used safely in all patients with the tachy-brady syndrome. Furthermore, Mun *et al*. demonstrated that unresponsiveness of SAN to sympathetic stimulation could be a mechanism of the SND induced by amiodarone^17^. Thus, ionic mechanisms underlying amiodarone-induced bradycardia under the AF-induced SND condition remain unclear.

Mathematical models of cardiac cells are widely considered as potentially important tools for safety pharmacology evaluation^18–20^. Rodriguez *et al*.^21^, Bottino *et al*.^22^, Mirams *et al*.^23^ and Davies *et al*.^24^ used ion channel data acquired from routine high-throughput screens to infer results during compound development. Pharmaceutical companies, the Comprehensive *in Vitro* Proarrythmia Assay (CiPA), the United States Food and Drug Administration (FDA) and the Cardiac Safety Research Consortium raised *in silico* drug assays to provide integrative, high-throughput, cost-effective and efficient solutions^25^. Prediction of arrhythmogenicity was improved by considering the effects of drugs on multiple ion channels, the therapeutic plasma drug concentrations and the use of biophysically detailed mathematical models of cardiac electrical activity^26^.

In the present work, based on a biophysically detailed mathematical model of human SAN cells^27^, we developed a human SND model by considering AF-induced electrical remodelling, which includes changes in membrane ionic currents and calcium handling. The ionic mechanisms of SND in patients with AF were investigated by assessing the role of each remodelled target in regulating SAN automaticity. The amiodarone effects were integrated into the SND cell model by adapting the ion channel conductivities to the dose-dependent inhibition of the currents. The impacts of amiodarone on voltage clock and autonomic regulation of SAN cells were investigated by assessing the roles of membrane ionic currents, calcium handling and sympathetic and/or parasympathetic stimulation, respectively. Furthermore, some antiarrhythmic agents (i.e., disopyramide, quinidine and digoxin) used for improving sinoatrial node function in the setting of AF-induced SND were assessed.

## Methods

This work was conducted based on a mathematical model and didn’t involve any experimental animals or human participants.

### Modelling AF-induced electrical remodelling underlying SND

The human SAN cell model developed by Fabbri *et al*.^27^ was used as the base model for single-cell simulations in this study since the model was based on and validated using electrophysiological data from isolated human SAN pacemaker cells. In the cellular model, an ordinary differential equation was used to describe the transmembrane potential *V*:

1 $$\frac{dV}{dt}=-\frac{{I}_{ion}}{{C}_{m}}$$

where *t* is the time, *C*_*m*_ (57 pF) is the capacitance across the cell membrane, and *I*_*ion*_ is the total ionic current across the membrane.

2 $${I}_{ion}={I}_{f}+{I}_{CaL}+{I}_{CaT}+{I}_{Na}+{I}_{Kr}+{I}_{Ks}+{I}_{to}+{I}_{Kur}+{I}_{KACH}+{I}_{NCX}+{I}_{NaK}$$

The precise behavior of the individual channels was based on a wide range of human cell electrophysiological data, and details can be found in the study conducted by Fabbri *et al*.^27^

**Figure 1:**
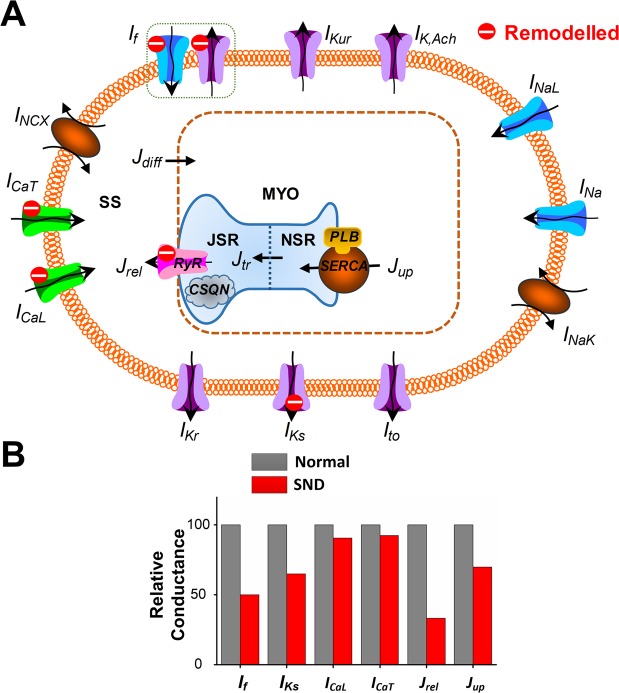
Illustration of atrial fibrillation (AF)-induced remodelling processes and electrophysiological properties and calcium dynamics in the human sinoatrial node (SAN) cell model. (**A**) Schematic presentation of the cell model. Formulations for AF-induced remodelled currents and fluxes (red) were based directly on experimental data of the canine model of pacing-induced AF. The model includes four compartments: bulk myoplasm (myo), junctional sarcoplasmic reticulum (JSR), network sarcoplasmic reticulum (NSR), and subspace (SS). Currents into the subspace: funny current (*I*_*f*_; representing both sodium and potassium components), L-type calcium current (*I*_*CaL*_), T-type calcium current (*I*_*CaT*_), fast sodium current (*I*_*Na*_), transient outward potassium current (*I*_*to*_), rapid delayed rectifier potassium current (*I*_*Kr*_), slow delayed rectifier potassium current (*I*_*Ks*_), ultrarapid delayed rectifier potassium current (*I*_*Kur*_), acetylcholine-sensitive muscarinic potassium current (*I*_*KACh*_), sodium-calcium exchange current (*I*_*NCX*_) and sodium-potassium pump current (*I*_*NaK*_). Ionic fluxes: calcium flux (*J*_*rel*_) through ryanodine receptor (RyR), NSR to JSR calcium translocation (*J*_*tr*_), calcium uptake (*J*_*up*_) into NSR via SERCA/phospholamban (PLB) and diffusion calcium flux from subspace to myoplasm (*J*_*diff*_). AF-induced targets: *I*_*f*_, *I*_*CaT*_, *I*_*CaL*_, *J*_*rel*_ R and *J*_*up*_. (**B**) Relative conductance between normal and SND conditions.

Under the SND conditions, AF-induced electrical remodelling included voltage clock-associated ionic currents and calcium clock-associated calcium handling. The ionic current formulations were modified based on data from Yeh *et al*., who investigated the voltage clock of single SAN cells from dogs that underwent atrial tachypacing and measured SAN transcript expressions for *I*_*f*_ –associated subunits, *I*_*K*_-related subunits and *I*_*Ca*_ subunits^6^. Alterations in calcium handling properties were derived from experimental data of Joung *et al*., who evaluated the calcium clock of single SAN cells in pacing-induced AF dogs, and determined expression of RyR, SERCA and phospholamban (PLB)^9^. In the Fabbri *et al*. model, *I*_*f*_, *I*_*CaL*_, *I*_*CaT*_, *I*_*Ks*_, *J*_*rel*_ and *J*_*up*_ were decreased to 50%, 90%, 92%, 65%, 33% and 71%, respectively, for describing the AF-induced SND condition (Fig. 1A,B and Supplementary Table 1).

### Modelling effects of drugs on SND

In the present study, we focused on investigating the effects of amiodarone on SAN automaticity. The effects of amiodarone were incorporated into the cellular model by modifying ionic currents, involving beta-adrenergic receptor and membrane targets. For the block effects of amiodarone on the beta-adrenergic receptor, we decreased the effects of ISO stimulation by 15.2%^28^. For the effects of amiodarone on membrane targets, we integrated the block effects of amiodarone on funny current (*I*_*f*_)^29^, sodium currents (*I*_*Na*_)^30^, calcium currents (*I*_*CaL*_^28^ and *I*_*CaT*_^31^), potassium currents (*I*_*to*_^32^*, I*_*Kr*_^28^, *I*_*Ks*_^33^, *I*_*Kur*_^34^ and *I*_*KACh*_^35^), sodium/calcium exchange current *I*_*NCX*_^36^, and sodium/potassium pump current *I*_*NaK*_.^37^ Block of ionic currents provoked by amiodarone was simulated by including the fraction of unblocked channels in their formulations, estimated using the standard sigmoid dose-response curve parametrized using the half-maximal inhibitory concentration (*IC*_50_) and Hill coefficient (*nH*)^26^. Within the framework of pore block theory, the maximal conductance *g*_*i*_ of an ionic current type *i* was modified in a concentration-dependent manner, such that

3 $${g}_{i}={g}_{control,i}\frac{1}{1+{({[I{C}_{50}]}_{i}/D)}^{nH}}$$

where *g*_*control,i*_ represents the maximal conductance of the *i* current channel in drug-free conditions and *D* is the drug concentration. According to clinical data, the therapeutic concentration range of amiodarone, disopyramide, quinidine and digoxin, respectively, was 0.77~3.88 μM^38–43^, 6~15 µM^44^, 4~17 μM^45^ and 0.64~2.56 nM^46^. Therefore, *D* for amiodarone, disopyramide, quinidine and digoxin was set to be 1.55 μM, 10 µM, 4 μM and 1 nM, respectively. In addition, the concentration-dependent effects of drugs were also investigated at the low, middle, and high concentrations (Supplementary Table 2). The *IC*_50_ and *nH* values describing the effects of drugs on ionic currents were listed in the Supplementary Tables 3–6.

### Autonomic modulation

According to the method of Fabbri *et al*.^27^, autonomic regulation of SAN cells was studied by simulating the effects of acetylcholine (ACh, 10 nM) and isoprenaline (ISO, 1.0 μM) stimulations. Changes in *I*_*f*_, *I*_*CaL*_, *I*_*Ks*_, *I*_*KACh*_, *I*_*NaK*_ and *J*_*up*_ due to ACh and ISO stimulations were listed in the Supplementary Table 7.

### Multicellular 1D simulations

A 1D SAN-atrial model, which consists of 30 SAN cells and 60 atrial cells, was developed to simulate electrical waves under the AF-induced SND condition in the presence of drugs. In the 1D cable model, the mathematical model of the human atrial myocyte developed in our previous study^47–52^ was used, and AF was simulated by introducing electrical remodelling based on experimental data^53–62^ (listed in the Supplementary Table 8). The membrane potential is described by:

4 $$\frac{dV(i)}{dt}=\frac{{G}_{gap}(V(i-1)+V(i+1)-2V(i))-{I}_{ion}}{{C}_{m}}$$

where *V*(*i*) is the membrane potential of the *i*^th^ cell, *t* is time, *I*_*ion*_ is the sum of the transmembrane ionic currents, *C*_*m*_ is the total membrane capacitance and *G*_*gap*_ is the gap-junction coupling, which is given by:

5 $${G}_{gap}=\frac{1}{0.35+{F}_{cell}(1-0.35)}$$

The heterogeneity of the SAN was implemented in the model following the strategies of Zhang *et al*.^63^ and Garny *et al*.^37^ The method uses the parameters of the central and the peripheral cell to determine the characteristics of transitional cells. A scaling factor *F*_*cell*_ is calculated by:

6 $${F}_{cell}=\frac{1.07\cdot (i-0.1)}{(1.0+0.7745\cdot \exp (2.05-i)/0.295))}$$

with the location *i* of cells (*i*=1 central; *i*=30 peripheral). For 60 atrial cells, *C*_*m*_ = 1 *pF* and *G*_*gap*_ = 400 nS/pF.

### Simulation protocol and data analysis

To quantitatively assess the sensitivity of action potential (AP) and calcium features to parameters affected by electrical remodelling/drug actions, we changed values of parameters associated with each target between 100% to *x*%. *x* was set to be the minimal value to generate AP. Maximum diastolic potential (MDP), diastolic depolarization rate (DDR_100_), maximum voltage of AP (OS), maximum rate of rise of membrane potential (dV/dt)_max_, maximum value of intracellular calcium concentration (Cai_max_), minimum value of intracellular calcium concentration (Cai_min_) and heart rate were used to quantify electrophysiological characteristics of SAN myocytes. And electrophysiological features of atrial cells were quantified by measuring MDP, OS, (dV/dt)_max_, Cai_max_, Cai_min_ and AP duration at 90% repolarization (APD_90_). An explicit Euler method for solving the ordinary differential Eq. (4) with a time step of 0.00001 s was used. Simulations were run until steady-state was reached after 100 s (~10000000 times). For 1D simulations, it took about 5 h using the Intel Core I5–4210m Processor (3 M Cache, up to 3.20 GHz) to compute 100 s.

## Results

### Effects of AF-induced electrical remodeling on electrophysiological properties in SND

**Figure 2:**
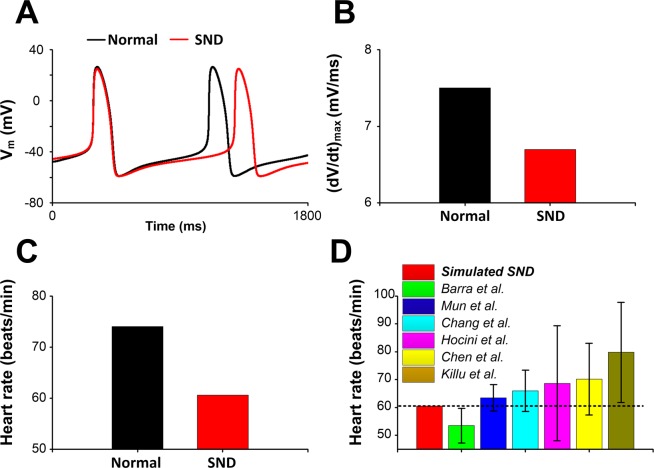
Action potentials (V_m_) and the heart rate of the human sinoatrial node (SAN) cell. (**A,B**) Comparison of action potentials (V_m_) and maximum rate of rise of membrane potential ((dV/dt)_max_) between normal and SND conditions. (**C**) AF-induced electrophysiological remodelling slowed the heart rate. (**D**) Simulated results are compared with the clinical findings (Barra *et al*.^64^, Mun *et al*.^17^, Chang *et al*.^65^, Hocini *et al*.^66^, Chen *et al*.^67^ and Killu *et al*.^68^) in patients with SND after catheter ablation of AF.

Single-cell simulations were performed under normal and SND conditions and effects of AF-induced electrical remodeling on AP were investigated (Fig. 2). Note that due to AF-induced electrical remodelling, the SND AP had a decrease in OS from 26.4 mV to 24.9 mV, a more gradual transition from phase 4 to phase 0 (DDR_100_, from 56.7 mV/s to 46.4 mV/s) (Fig. 2A) and a small (dV/dt)_max_ (Fig. 2B). Compared with the normal condition, the heart rate in the SND case decreased from 74 to 60 beats/min (Fig. 2C), which was consistent with clinical findings^17,64–68^ (Fig. 2D).

**Figure 3:**
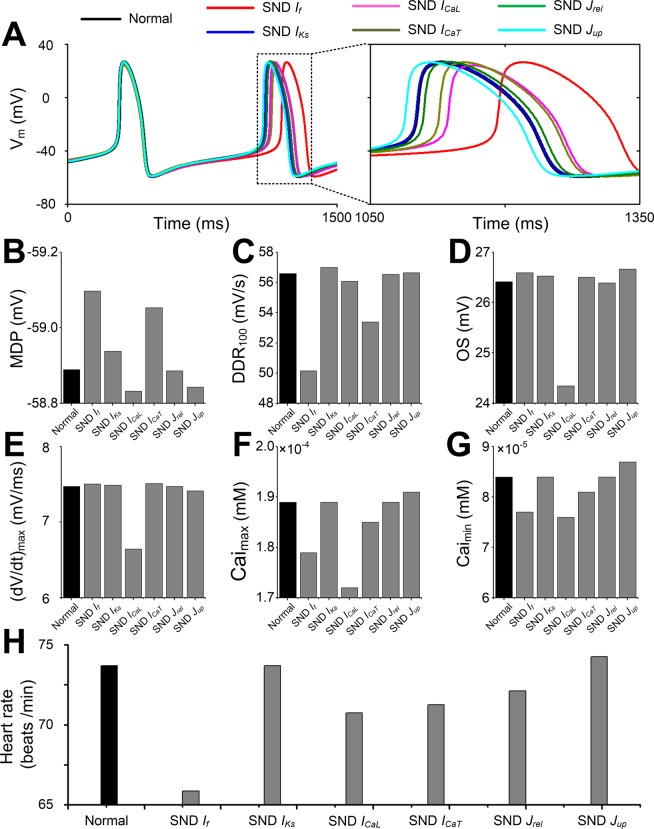
Effects of individual remodelling targets under sinus node dysfunction (SND) on action potential (AP) and the heart rate. (**A**) Action potential, (**B**) MDP, (**C**) DDR_100_, (**D**) OS, (**E**) (dV/dt)_max_, (**F**) Cai_max_, (**G**) Cai_max_ and (**H**) heart rate between normal (black) and remodelled sinoatrial node cells. Six remodelled cellular components (funny current, *I*_*f*_; slow delayed rectifier potassium current, *I*_*Ks*_; L-type calcium current, *I*_*CaL*_; T-type calcium current, *I*_*CaT*_, calcium flux through ryanodine receptor (*J*_*rel*_) and SERCA (*J*_*up*_)), are shown, respectively.

To investigate the role of each remodeled ionic current/flux under the SND condition, a series of control simulations with individual ionic remodeling were performed. AP (Fig. 3A), MDP (Fig. 3B), DDR_100_ (Fig. 3C), OS (Fig. 3D), (dV/dt)_max_ (Fig. 3E), Cai_max_ (Fig. 3F), Cai_min_ (Fig. 3G) and heart rate (Fig. 3H) of remodeled SAN cells were compared with AP features in normal cells. A more detailed analysis of the AP features reveals that changes in MDP and Cai_min_ were almost negligible for all remodelled conditions. DDR_100_ and Cai_max_ showed a substantial decrease for the SND *I*_*f*_ condition, whereas OS, (dV/dt)_max_ and Cai_max_ showed a reduction for the SND *I*_*CaL*_ condition. Of note, a significant reduction in heart rate (from 74 to 65.8 beats/min) was only observed in the SND *I*_*f*_ condition, but not under other remodeled conditions.

### Effects of amiodarone on ionic currents/fluxes of the human sinus node

**Figure 4:**
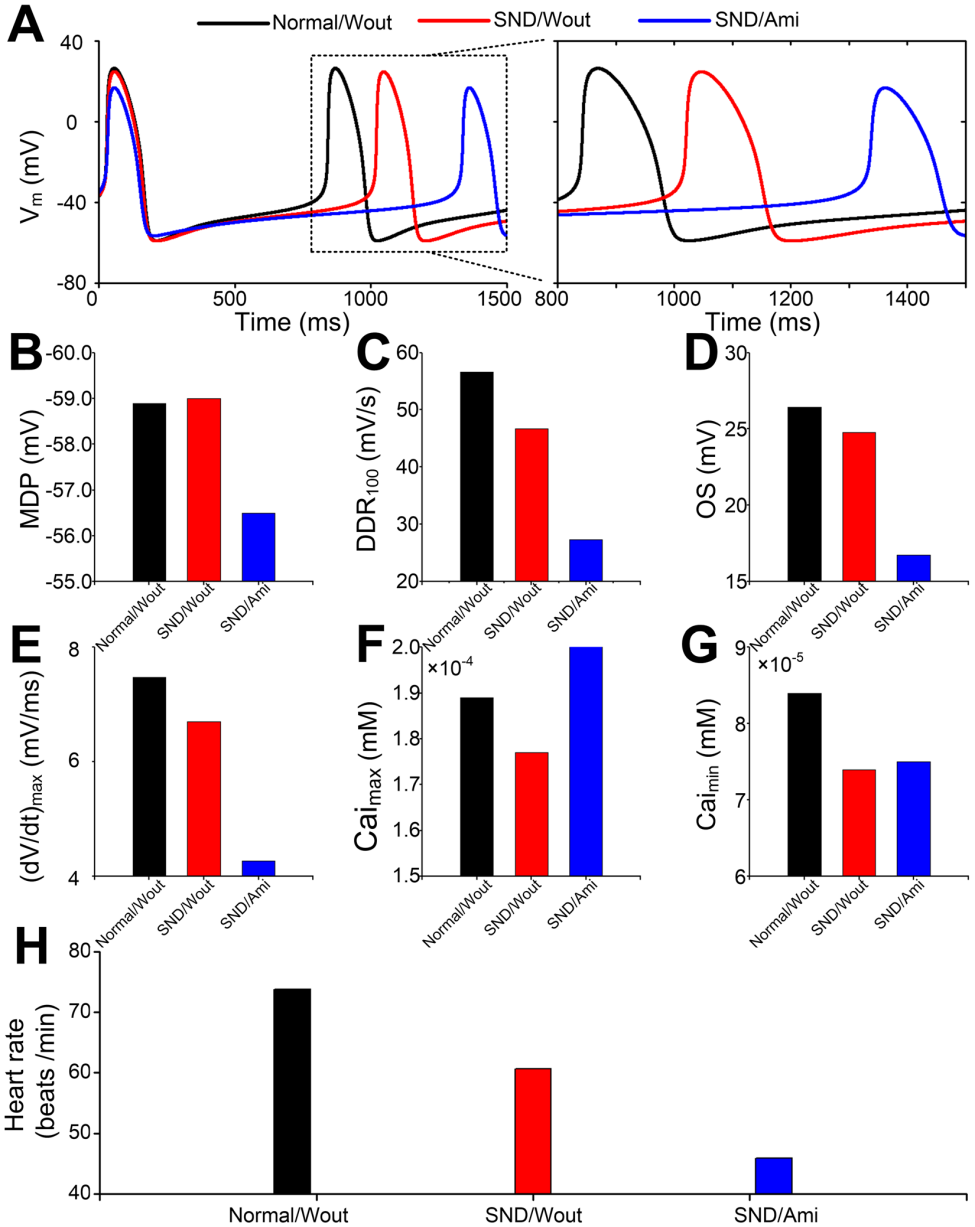
Effects of amiodarone on action potential (AP) and the heart rate under the sinus node dysfunction (SND) condition. (**A**) Action potential, (**B**) MDP, (**C**) DDR_100_, (**D**) OS, (**E**) (dV/dt)_max_, (**F**) Cai_max_, (**G**) Cai_max_ and (**H**) heart rate between normal without amiodarone (Normal/Wout), SND without amiodarone (SND/Wout) and SND with amiodarone (SND/Ami) cells.

To investigate the effects of amiodarone on SAN function in patients with AF, we simulated APs (Fig. 4A) and computed heart rate using the SND model by including the actions of amiodarone on ionic currents. In the presence of amiodarone, MDP was increased from -58.99 mV for the SND condition to -56.50 mV (Fig. 4B), DDR_100_, OS and (dV/dt)_max_ were reduced (Fig. 4C–E), and Cai_max_ and Cai_min_ were slightly increased (Fig. 4F,G). The model also predicted a decrease in the heart rate (Fig. 4H).

**Figure 5:**
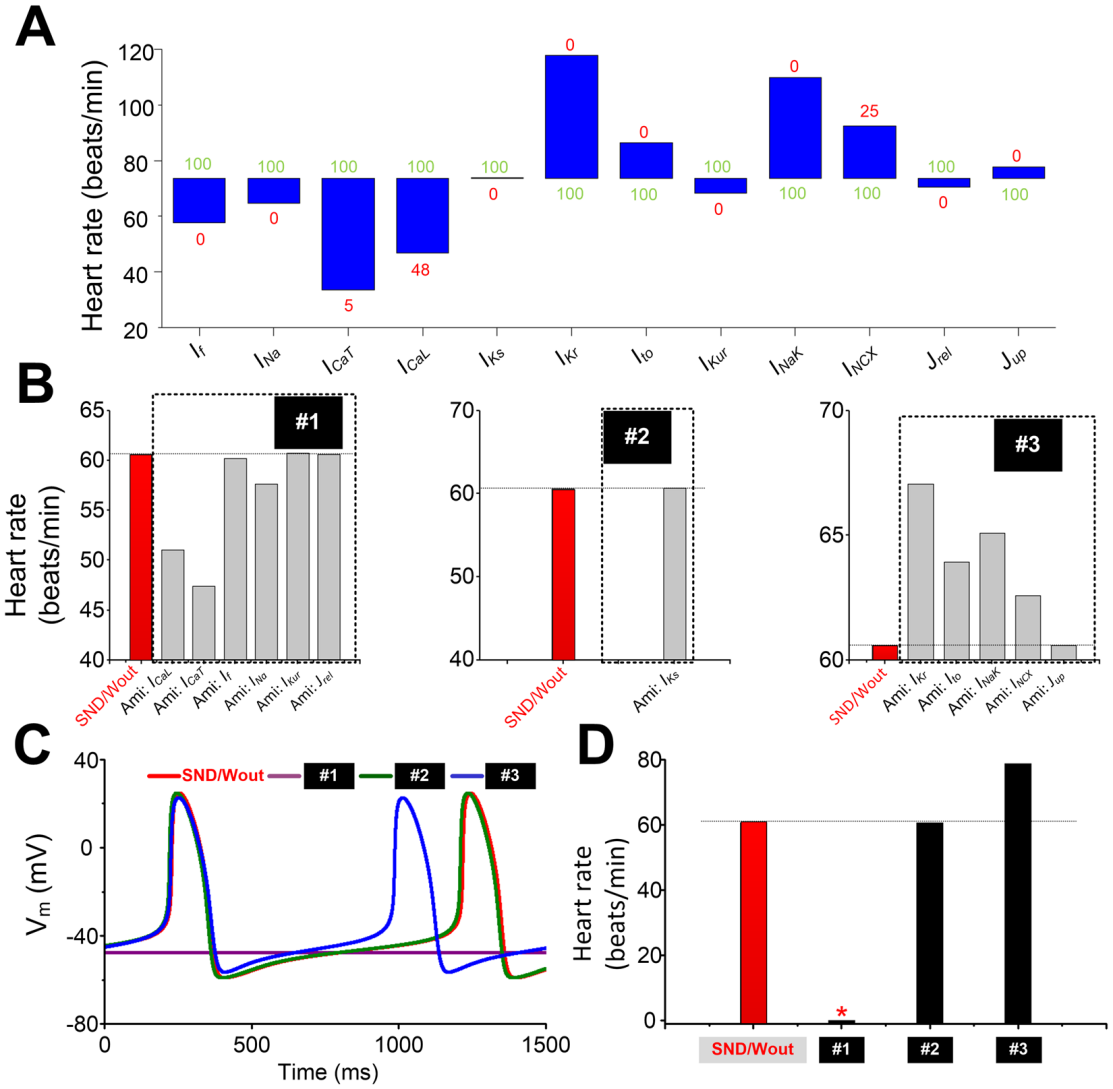
Effects of amiodarone on ionic currents/fluxes and consequent changes in heart rate. (**A**) Changes in heart rate when each ion current is reduced from 100% (green number) to *x*% (red number) in SAN cells. (**B**) Heart rate of sinus node dysfunction (SND) cells in the presence of amiodarone action on each target only. Inhibitions of *I*_*CaL*_, *I*_*CaT*_, *I*_*f*_, *I*_*Na*_, *I*_*Kur*_ and *J*_*rel*_ cause reductions in the heart rate, inhibitions of *I*_*Ks*_ have no effect on the heart rate, and inhibitions of *I*_*Kr*_, *I*_*to*_, *I*_*f*_, *I*_*NaK*_, *I*_*NCX*_ and *J*_*up*_ lead to increases in the heart rate. Group one (#1): *I*_*CaL*_, *I*_*CaT*_, *I*_*f*_, *I*_*Na*_, *I*_*Kur*_ and *J*_*rel*;_ group two (#2): *I*_*Ks*_; and group three (#3): *I*_*Kr*_, *I*_*to*_, *I*_*f*_, *I*_*NaK*_, *I*_*NCX*_ and *J*_*up*._ (**C-D**) Action potentials (APs) and the heart rate of SND cells in the presence of AMI action on targets in the group #1, #2 and #3. The Red star indicates heart arrest.

To quantitatively assess the sensitivity of pacing rate to each target (*I*_*CaL*_, *I*_*CaT*_, *I*_*f*_, *I*_*Na*_, *I*_*Kur*_, *I*_*Ks*_, *I*_*Kr*_, *I*_*to*_*, I*_*NaK*_*, I*_*NCX*_*, J*_*rel*_ or *J*_*up*_) of AF-induced remodelling and amiodarone actions, we carried out experiments by reducing each ionic current/flux from 100% to *x* % (the minimal value to generate AP). As is shown in Fig. 5A, a reduction in heart rate in blocking *I*_*CaL*_, *I*_*CaT*_, *I*_*f*_, *I*_*Na*_, *I*_*Kur*_, or *J*_*rel*_ condition, an increase in heart rate in the case of inhibiting *I*_*Kr*_, *I*_*to*_*, I*_*NaK*_, *I*_*NCX*_ or *J*_*up*,_ and no significant changes (i.e., changes in heart rate are no more than one beat) in heart rate under blocking *I*_*Ks*_ condition are observed (Supplementary Table 9). Based on the role of each ionic current/flux modulated by amiodarone in regulating heart rate, ionic currents were grouped into three categories: #1 (*I*_*CaL*_, *I*_*CaT*_, *I*_*f*_, *I*_*Na*_, *I*_*Kur*_ and *J*_*rel*_), #2 (*I*_*Ks*_) and #3 (*I*_*Kr*_, *I*_*to*_*, I*_*NaK*_*, I*_*NCX*_ and *J*_*up*_). Figure 5B showed changes in heart rate when each ionic current/flux was blocked only in the presence of 1.55 µM amiodarone. The combined effect of amiodarone on AP was further investigated in each of the groups (Fig. 5C), and the respective heart rate was 0, 60.63 and 78.81 beats/min, respectively (Fig. 5D). Thus, opposing effects were present. However, the combined actions of targets in the #1 far outweighed the effects of amiodarone on membrane targets in the #3.

### Effects of amiodarone on autonomic modulation of the human sinus node

**Figure 6:**
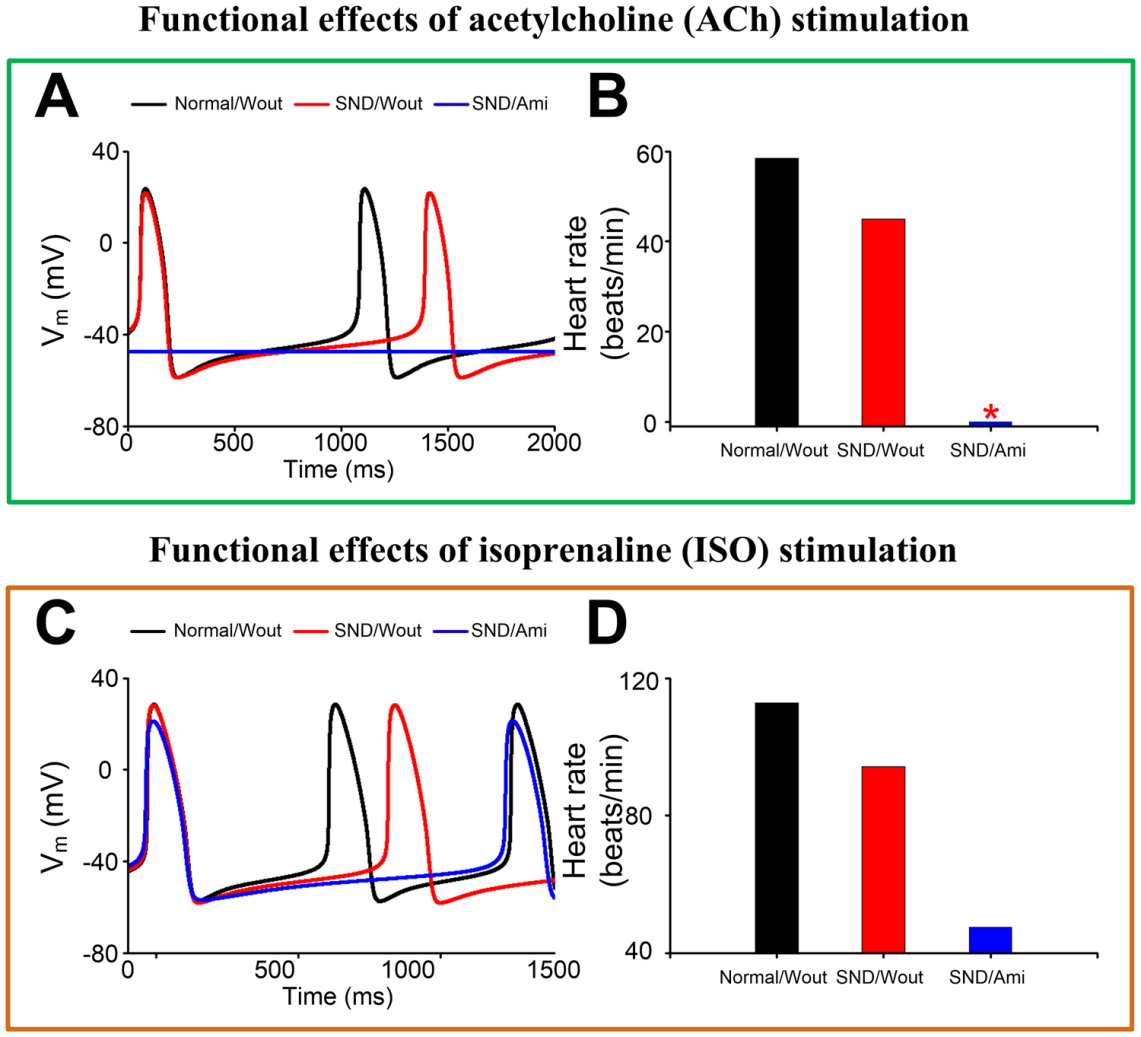
Functional effects of acetylcholine (ACh) and isoprenaline (ISO) stimulation. Effects of ACh on membrane potential (**A**, V_m_) and the heart rate (**B**) in the normal without amiodarone (Normal/Wout), SND/Wout and SND with amiodarone (SND/Ami) conditions. Effects of ISO on membrane potential (**C**, V_m_) and the heart rate (**D**).

To investigate the effects of amiodarone on the autonomic modulation of human SAN in patients with AF, we simulated APs of SAN cells with ACh and ISO stimulation under the normal without amiodarone (Normal/Wout), SND/Wout and SND with amiodarone (SND/Ami) conditions. After the administration of ACh, the heart rate for Normal/Wout and SND/Wout cells was 58.40 and 45.03 beats/min, respectively. The amiodarone actions further reduced the heart rate and caused heart arrest under the SND/Ami condition (Fig. 6A,B). After the administration of ISO, the heart rate for Normal/Wout and SND/Wout cells was 112.72 and 94.23 beats/min, respectively. With the addition of amiodarone on top of ISO, the heart rate was reduced to 47.47 beats/min under the SND/Ami condition (Fig. 6C,D).

### Effects of disopyramide, quinidine and digoxin on the human sinus node

**Figure 7:**
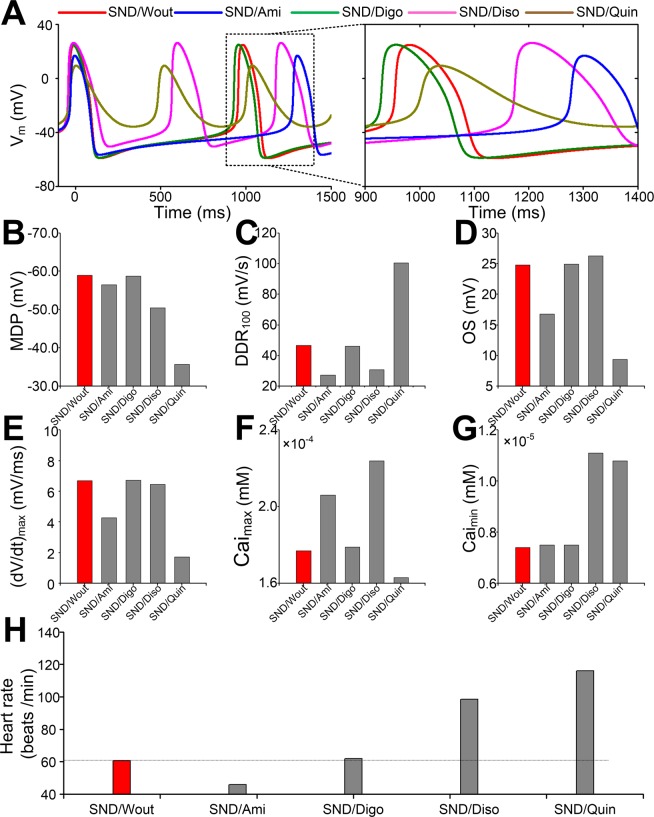
Effects of amiodarone (Ami), digoxin (Digo), disopyramide (Diso) and quinidine (Quin) on action potential (AP) and the heart rate in sinus node dysfunction (SND). (**A**) AP, (**B**) MDP, (**C**) DDR_100_, (**D**) OS, (**E**) (dV/dt)_max_, (**F**) Cai_max_, (**G**) Cai_max_ and (**H**) heart rate under the SND condition (SND/Wout) are compared with SND in the presence of amiodarone (SND/Ami), digoxin (SND/Digo), disopyramide (SND/Diso) and quinidine (SND/Quin), respectively.

To examine whether there are antiarrhythmic drugs that have efficacy for the treatment of AF-induced SND, we investigated the effects of amiodarone (Ami), digoxin (Digo), disopyramide (Diso) and quinidine (Quin) on SAN function (Fig. 7). Compared with the drug-free SND condition (SND/Wout), there was an increase in cycle length in the presence of amiodarone, but a reduction in cycle length was observed in the presence of digoxin, quinidine and disopyramide (Fig. 7A). In the presence of disopyramide (SND/Diso), MDP, OS, Cai_max_ and Cai_min_ were slightly increased, whereas (dV/dt)_max_ had almost no changes and DDR_100_ was slightly reduced. In the presence of quinidine (SND/Quin), MDP, DDR_100_ and Cai_min_ were increased, whereas OS, (dV/dt)_max_ and Cai_max_ were significantly reduced. In the presence of digoxin (SND/Digo), all biomarkers had almost no changes (Fig. 7B–G). The heart rate was reduced from 60.62 to 45.94 beats/min in the presence of amiodarone, whereas the heart rate was increased from 60.62 to 61.79, 98.60 and 116.03 beats/min, respectively, in the presence of digoxin, disopyramide and quinidine (Fig. 7H).

The concentration-dependent effects of these drugs were also investigated at the low, middle, and high concentrations. Compared with the drug-free SND condition (SND/Wout), heart arrest was observed in the presence of amiodarone (SND/Ami(High)) or quinidine (SND/Quin(High)) at the high concentration, an increase in heart rate was predicted in the presence of disopyramide, and no significant changes in heart rate were shown in the presence of digoxin (Supplementary Fig. 1).

Further simulations were conducted to examine whether these antiarrhythmic drugs have efficacy for improving the SAN function using a 1D SAN-atrial model. Compared with the normal condition (Supplementary Fig. 2A), the number of electrical waves within 5 s reduced from 7 to 5 beats under the SND condition without any drugs (Supplementary Fig. 2B). In the presence of drugs, the number of electrical waves under the SND condition showed an increase in the presence of disopyramide (Supplementary Fig. 2C), no significant changes in the presence of digoxin (Supplementary Fig. 2D), a reduction in the presence of amiodarone (Supplementary Fig. 2E) and SAN arrest in the presence of quinidine (Supplementary Fig. 2F).

## Discussion

In the present study, we formulated a human AP model of AF-induced SND and investigated the impact of amiodarone on human SAN function. We further assessed whether other drugs (i.e., disopyramide, quinidine and digoxin) could reverse the AF-induced SND phenotype. The major findings of this study are as follows: (1) the AF-induced SND can be mainly attributed to down-regulation of *I*_*f*_; (2) the effects of amiodarone lead to a lower DDR_100_ and more prolonged diastolic depolarization phase, resulting in a slower pacemaking rate and contributing to the impact of amiodarone on human SAN function; (3) the bradycardiac effects of amiodarone are likely to be amplified by vagal nerve activity (simulated addition of ACh to the SND cells with amiodarone causes SAN arrest); and (4) our model predicted an increase in pacemaking rate in the presence of disopyramide. Together, these data point to voltage-clock dysfunction underlying SND and provide evidence substantiating the impact of amiodarone on the function of the SAN.

The leading causes of bradycardia under the AF-induced SND are electrophysiological remodelling related to the voltage clock of the human SAN. Previous experiments on canine SAN cells have demonstrated that AF-induced remodelling of ion channels, particularly for the “pacemaker” subunit *I*_*f*_, may contribute to the clinically significant association between SND and AF^6^. Our simulated effects of remodelled *I*_*f*_ are concordant with this experimental findings^33^. In additional to remodelled *I*_*f*_, changes in *I*_*Ks*_, *I*_*CaL*_ and *I*_*CaT*_ were observed in SAN cells of the pacing-induced AF canine model^69^. We assumed that a similar ionic remodelling may also occur in human AF-induced SND. The combined effect of the electrical remodelling slowed down the heart rate significantly. The reduction in heart rate was mainly the result of a lower DDR_100_ arising from the downregulation of inward currents (including *I*_*f*_, *I*_*CaL*_ and *I*_*CaT*_). And remodelled *I*_*Ks*_ has a negligible effect on DDR_100_ and the pacemaking rate. These findings agree with previous work showing the strong contributions of *I*_*f*_, *I*_*CaL*_ and *I*_*CaT*_ to DDR_100_ and the pacing rate^27^. The sensitivity analysis highlighted the strong impact of *I*_*CaL*_ on heart rate. 52% block of *I*_*CaL*_ is able to lead to heart arrest, whereas 95% block of *I*_*CaT*_ results in hart arrest and a slow heart rate is obtained with 100% block of *I*_*f*_ (Fig. 5A). The illustrative investigation of the effects of changes in maximal conductances of ionic current suggests that *I*_*CaL*_ plays a role in pacemaking, which is consistent with the sensitivity analysis by Fabbri *et al*.^27^. Under the SND condition, *I*_*CaL*_, *I*_*CaT*_ and *I*_*f*_ were decreased to 90%, 92% and 50%, respectively (Fig. 1B). Remodelled *I*_*CaL*_ and *I*_*CaT*_ caused a reduction in heart rate from 74 to 70.7 and 71.2 beats/min, respectively, whereas remodelled *I*_*f*_ led to a slower heart rate from 74 to 65.8 beats/min. Therefore, the reduced *I*_*f*_ under the SND condition mainly contributed to the heart rate reduction.

**Figure 8:**
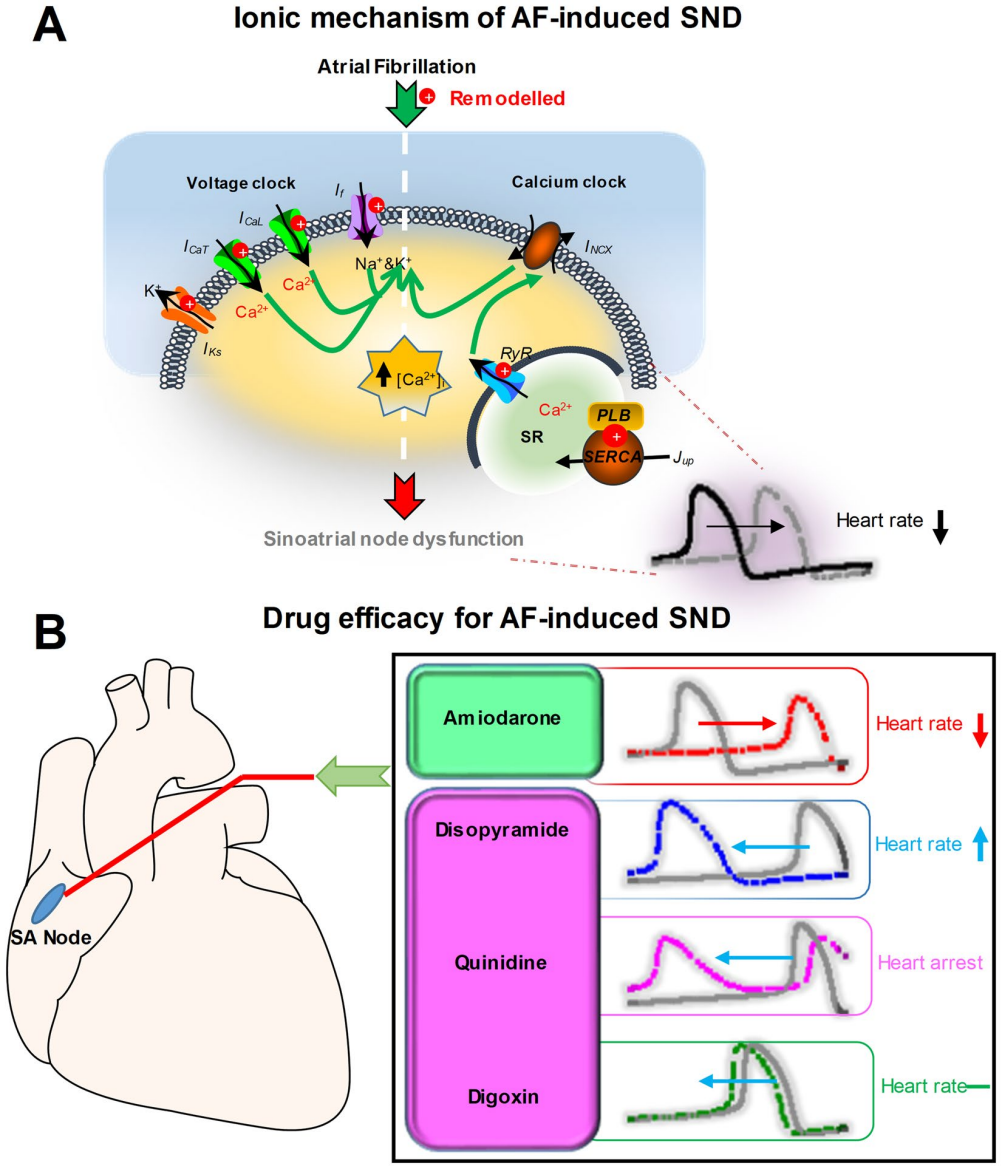
Ionic mechanisms of atrial fibrillation (AF)-induced sinoatrial node dysfunction (SND) and drug efficacy for AF-induced SND. (**A**) Atrial fibrillation-induced electrical remodelling includes *I*_*f*_, *I*_*CaL*_, *I*_*CaT*_, *I*_*Ks*_, *J*_*rel*_ and *J*_*up*_*. I*_*f*_, *I*_*CaL*_ and *I*_*CaT*_ in combination may be responsible for the diastolic depolarization, the so-called “voltage clock”. Diastolic spontaneous calcium release via *RyR* increases the calcium concentration ([Ca^2+^]_i_) and activates sodium-calcium exchanger. The inward *I*_*NCX*_ contributes to the diastolic depolarization, the so-called “calcium clock”. Therefore, AF-induced electrical remodelling impairs both the voltage clock and the calcium clock, resulting in the heart rate reduction and SND. (**B**) Under the AF-induced SND condition, the action of amiodarone impairs sinoatrial node function, leading to a reduction in the sinus rate, whereas disopyramide may improve sinoatrial node function and increase the sinus rate.

Previous experiments have shown that AF-induced SND is also associated with calcium handling abnormalities. Here, we also investigated the effects of calcium handling properties on SAN automaticity. AF-induced changes in calcium handling (the downregulation of *J*_*rel*_ and *J*_*up*_) in our human SAN model are similar to experimental data from a canine model of pacing-induced AF^9^. Downregulation of *J*_*rel*_ could decrease the pacemaking rate (from 73.7 to 72.1 beats/min) which is consistent with experimental observations^9^, whereas remodelled *J*_*up*_ could increase heart rate (from 73.7 to 74.2 beats/min) which is a good agreement with previous modelling study^27^. However, changes in the pacemaking rate due to calcium handling abnormalities were almost negligible. Altogether, our simulation results indicate that voltage- clock malfunction might be the mechanism underlying AF-induced SND (Fig. 8A) and our SND mathematical model for human SAN cells can be useful in the design of experiments and the development of drugs.

Amiodarone is the most frequently used agent to reverse AP shortening (Supplementary Fig. 3) in patients with AF, which is partly resulted from electrical remodelling^70^ (Supplementary Table 10). However, computational drug treatment simulations predicted a dramatical reduction in the pacemaking rate, indicating the impact of amiodarone on the SAN function. These findings agree with the previous work showing amiodarone-induced bradycardia in AF patients^71^. These changes in the pacemaking rate may be attributable to the effects of amiodarone on multiple ion channels and beta-adrenergic receptors. Inhibition of *I*_*Na*_*, I*_*Kur*_*, I*_*CaL*_*, I*_*CaT*_*, I*_*f*_*, J*_*rel*_ and beta-adrenergic receptors leads to a slower heart rate, whereas the block of *I*_*Kr*_*, I*_*to*_*, I*_*KACh*_*, I*_*NaK*_, *I*_*NCX*_ and *J*_*up*_ causes a higher heart rate. Thus, opposing effects were present. However, deceleration effects of amiodarone far outweighed its acceleration effects, leading to a slow pacemaking rate. In addition, the slow heart rate since the actions of amiodarone on *I*_*CaL*_ in the current study in accordance with the work of Nattel *et al*.^72^, suggesting that changes in SAN function can be attributable to amiodarone’s calcium channel-blocking properties and account for the adverse consequence of amiodarone.

Our study substantiates the notion that beta-adrenergic blocking effects of amiodarone may explain the unresponsiveness of SAN to sympathetic stimulation in AF patients^17^. Reports of AF patients suggest that amiodarone causes SND, which results in reduced P-wave amplitude at baseline and during ISO infusion. The anti-adrenergic effects of amiodarone showed a reduction of receptor density in the cellular membrane, suggesting that amiodarone leads to the unresponsiveness of SAN to ISO stimulation^73^. Moreover, the overall pacing rate acceleration because of the administration of 1 µM ISO was the result of a balance between opposing contributors. Previous modelling studies have demonstrated that in five targets (*I*_*f*_*, I*_*CaL*_*, I*_*NaK*_*, I*_*Ks*_ and *J*_*up*_) ISO-induced changes occur in *I*_*f*_ and *I*_*CaL*_, leading to a faster heart rate^27^. However, amiodarone has block effects on both *I*_*f*_ and *I*_*CaL*_. And our simulated results show that the SAN pacing rate decreases in the presence of amiodarone. These studies and our results suggest that amiodarone causes bradycardia by partly inhibiting *I*_*f*_, *I*_*CaL*_ and beta-adrenergic receptors in the human SAN. Thus, our results suggest that amiodarone cannot be used safely in patients that have SND associated with AF. We further investigated effects of quinidine, disopyramide and digoxin on the function of the SAN under the AF-induced SND condition and simulated results demonstrated that disopyramide was necessary to considerably increase the heart rate (Fig. 8B).

Several limitations specific to this study are addressed here. Firstly, the electrophysiological representation of AF-induced remodelling in the human SND model is based on data from previous canine models of AF^6,9^, however, because of the lack of experimental data on humans. Special attention must be paid to the differences between canine and human sinoatrial node^74^. Secondly, the blocking effects of drugs on ionic currents were modelled using the total plasma concentrations of drugs, nH and IC_50_ in the present study. However, drug efficacy needs to be related to free drug concentrations, not the total plasma concentrations^75^. Special attention should be paid to explain our simulated results, and free drug concentrations of drugs in plasma should be used to further assess the efficacy of drugs in the treatment of AF-induced SND. In addition, the large variability in IC_50_ was observed for most of the drugs, including amiodarone^76^. In a previous study, differential responses of ventricular and atrial ion channels to antiarrhythmic drugs were observed^77^. In the present study, IC_50_ values are chosen based on experimental data from atrial cells (where data are available) and large ventricular cells (where atrial data are not available). Thirdly, the uncertainty analysis is important for a more precise evaluation of the safety of antiarrhythmic drugs^25,78^. Therefore, our models and methodology should be improved, and statistics and treatment of uncertainties should be considered^78–80^ and further investigated. Fourthly, the block effects of amiodarone on beta-adrenergic receptors were modelled with the same percentage decrease in the effects of beta-adrenergic receptor stimulation on targets. The strict linearity is unlikely and the modelling approach should be improved based on experimental data. Fifthly, simulated results demonstrated that inhibition of *I*_*Kr*_ increases heart rate, but experimental studies showed that dofetilide (an *I*_*Kr*_ blocker) depolarized the maximum diastolic potentials, reduced the slope of the pacemaker potential and then abolished spontaneously firing action potentials in the nodal cells, suggesting *I*_*Kr*_ blockers slowed spontaneous activity^81,82^. The *I*_*Kr*_ model of Fabbri *et al*. model should be modified and verified based on these experimental data. Finally, the coupling between calcium clock and voltage clock is limited in the Fabbri *et al*. model, but the coupled-clock mechanism was established in two mathematical models by Kharche *et al*.^83^, and Maltsev and Lakatta^84^. Therefore, the Fabbri *et al*. model should be improved and calcium clock under the AF-induced SND condition should be further investigated.

In this study, we presented a simulation study investigating the effects of the antiarrhythmic agents in the setting of AF-induced SND. On the basis of the simulations, it can be concluded that amiodarone may impair the function of the SAN by slowing the heart rate whereas it has an antiarrhythmic effect on AF by prolonging AP. Additionally, disopyramide can reverse the AF-induced SND phenotype by increasing the heart rate. Therefore, disopyramide may be a desirable choice under the AF-induced SND condition.

## Supplementary information

Supplementary Information.
