# Supplementary material for: In silico study of the effects of anti-arrhythmic drug treatment on sinoatrial node function for patients with atrial fibrillation
Source: Sci Rep. 2020 Jan 15;10:305. doi: 10.1038/s41598-019-57246-5 (PMC6962222; doi:10.1038/s41598-019-57246-5)
Supplement: Supplementary file 1 — Supplementary Information. [file 41598_2019_57246_MOESM1_ESM.docx]

***In silico* study of the effects of anti-arrhythmic drug treatment on sinoatrial node function for patients with atrial fibrillation**

Jieyun Bai^1*^, Yaosheng Lu^1^ and Henggui Zhang^2*^

^1^Department of Electronic Engineering, College of Information Science and Technology, Jinan University, Guangzhou, China

^2^Biological Physics Group, School of Physics & Astronomy, University of Manchester, United Kingdom

^*^Corresponding author

E-mail: [bai_jieyun@126.com](mailto:bai_jieyun@126.com) (JB) and henggui.zhang@manchester.ac.uk (HZ)

1. **Simulation of atrial fibrillation (AF) and AF-induced sinoatrial node dysfunction (SND)**

The Fabbri et al. human SAN cell model was used as the base model for single-cell simulations^1^. SND was simulated by incorporating AF-induced electrical remodelling. According to experimental data from Yeh et al^2^. and Joung et al^3^., changes to *I_f_*, *I_Ks_*, *I_CaL_*, *I_CaT_* and calcium handling properties are listed in the **Table 1**. Also, AF was simulated by introducing electrical remodelling^4^ into our atrial cell model^5^originated from the ten Tusscher-Panfilov (TP) model^6^. Changes to our atrial model are listed in the **Table 8.** The time derivative of the membrane potential *V* is given by

$C_{m}\frac{dV}{dt}={-(I}_{ion}+I_{stim})$ (1)

Where $I_{ion}$ and $I_{stim}$ are the total ionic current and stimulus current flowing across the membrane and $C_{m}$ is the total membrane capacitance. The total ionic current is given by

$$I_{ion}=I_{Na}+I_{CaL}+I_{to}+I_{Kur}+I_{Kr}+I_{Ks}+I_{K1}+I_{NCX}+I_{NaK}$$

$+I_{pK}+I_{pCa}+I_{Nab}+I_{Cab}$ (2)

As is shown in **Figure 3A,** action potential duration (APD) shortening is obtained under the AF condition.

To examine AP features with respect to parameters affected by electrical remodelling/drug actions, we changed values of parameters associated with each target between 100% to *x*% (*x* is the maximum value of changes in each target). Maximum diastolic potential (MDP), heart rate, maximum rate of rise of membrane potential [(dV/dt)_max_], AP duration at 90% repolarization (APD_90_), maximum voltage of AP (OS), diastolic depolarization rate (DDR_100_), (Cai)_min_ and (Cai)_max_ were used to quantify the AP features (**Table 9** and **Table 10**). Inhibition of calcium currents (*I_CaT_* and *I_CaL_*) led to CL prolongation, whereas inhibition of *I_Kr_* caused CL shortening. Moreover, APD abbreviation in AF patients can be attributed to remodelled *I_Ks_* and *I_CaL_* **(Figure 3B)***.*

1. **Modelling effects of drugs**

The effects of amiodarone, disopyramide, quinidine and digoxin on SAN were investigated. The effects of these drugs were incorporated into the cellular model by modifying ionic currents, including membrane targets and calcium handling components, according to recent experimental data.

Changes of ionic currents for simulating actions of amiodarone, disopyramide, quinidine and digoxin, are listed in the **Table 3, Table 4, Table 5 and Table 6,** respectively.

The concentration-dependent effects of drugs were also investigated at the low, middle, and high concentrations (**Table 2**).

1. **1D simulations**

The 1D cable model consists of 30 SAN cells and 60 atrial cells. Current is described by:

$\frac{dV(i)}{dt}=\frac{{G_{gap}(V\left( i-1 \right)+V\left( i+1 \right)-2V\left( i \right))-I}_{ion}}{C_{m}}$ (3)

Where *V* is the membrane potential, *t* is time, *I_ion_* is the sum of the transmembrane ionic currents, $C_{m}$(57pF) is the total membrane capacitance and *G_gap_* is the gap-junction coupling, which is given by:

$G_{gap}=\frac{1}{0.35+F_{cell}\left( 1-0.35 \right)}$ (4)

The heterogeneity of the SAN was implemented in the model following the strategies of Garny et al.^7^ and Zhang et al^8^. The method uses the parameters of the central and the peripheral cell to determine the characteristics of transitional cells. A scaling factor $F_{cell}$ is calculated by :

$F_{cell}=\frac{1.07\cdot(i-0.1)}{(1.0+0.7745\cdot exp((2.05-i)/0.295)}$ (5)

with the location *i* of cells (*i*=1 central; *i*=30 peripheral). For 60 atrial cells, $C_{m}=1pF$ and $G_{gap}=400nS/pF$. The space step (0.1 mm) and the time step (0.00001s) were set to perform 1D simulations.

In the 1D simulations, compared with electrical waves under the SND condition, increased CL in the presence of disopyramide, no significant changes in the presence of digoxin, reduced CL in the presence of amiodarone and SAN arrest in the presence of quinidine.

1. **Supplementary Tables**

**Table 1. Changes of individual ionic currents from normal to atrial fibrillation-induced sinoatrial node dysfunction (SND) in the Fabbri et al. human sinoatrial node model.**

| Parameters | Current | Normal (%) | SND (%) | References based |
| --- | --- | --- | --- | --- |
| *I_f_* | Funny current | 100 | 50 | Yeh et al.^2^ |
| *I_CaL_* | L-type calcium current | 100 | 90 | Yeh et al.^2^ |
| *I_CaT_* | T-type calcium current | 100 | 92 | Yeh et al.^2^ |
| *I_Ks_* | Slow delayed rectifier potassium current | 100 | 65 | Yeh et al.^2^ |
| *J_rel_* | Sarcoplasmic reticulum calcium release flux | 100 | 33 | Joung et al.^3^ |
| *J_up_* | Sarcoplasmic reticulum calcium uptake flux | 100 | 71 | Joung et al.^3^ |

**Table 2. Drug concentrations at the low, middle and high concentrations.**

|  | Ami (µM) | Digo (nM) | Diso (µM) | Quin (µM) |
| --- | --- | --- | --- | --- |
| Low | 0.77 | 0.64 | 6.00 | 4.00 |
| Mid | 2.33 | 1.60 | 10.0 | 10.0 |
| High | 3.88 | 2.56 | 15.0 | 17.0 |

Abbreviations: Ami - amiodarone, Diso - disopyramide, Quin - quinidine and Digo – digoxin.

**Table 3. The effects of amiodarone with a concentration of 1.55 μM on ionic currents, transporters and exchangers.**

| Ionic currents | IC_50_ (μM) | nH | References based |
| --- | --- | --- | --- |
| *I_Na_* | 4.84 | 0.76 | Lalevée et al.^9^ |
| *I_f_* | 2.10 | 0.90 | Fan et al.^10^ |
| *I_CaL_* | 0.4-5.8 | 1.00 | Heijman et al.^11^ |
| *I_CaT_* | 2.40 | 1.00 | Yamashita et al.^12^ |
| *I_to_* | 4.90 | 1.00 | Varró et al.^13^ |
| *I_Ks_* | 3.84 | 0.63 | Kamiya et al.^14^ |
| *I_Kr_* | 10.0 | 1.00 | Heijman et al.^11^ |
| *I_Kur_* | 132.86 | 1.00 | Kobayashi et al.^15^ |
| *I_KACH_* | 2.00 | 1.33 | Watanabe et al.^16^ |
| *I_NCX_* | 3.60 | 1.00 | Watanabe et al.^17^ |
| *I_NaK_* | 15.6 | 1.00 | Gray et al.^18^ |
| *Beta receptor* | 8.70 | 1.00 | Heijman et al.^11^ |

Reduction of ionic currents (% of original value used in the computer model) due to the introduction of amiodarone. IC_50_ indicates the half-maximal inhibitory concentration; nH, Hill coefficient. Beta receptor blocking was modelled by reducing effects of ISO-stimulation (See details in Table 7).

**Table 4. The effects of disopyramide with a concentration of 10 μM on ionic currents.**

| Ionic currents | IC_50_ (μM) | nH | References based |
| --- | --- | --- | --- |
| *I_Kr_* | 10.66 | 1.07 | McPate et al.^19,20^ |
| *I_Na_* | 168.4 | 1.09 | Yasuda et al.^21^ |
| *I_to_* | 259 | 1.07 | Sanchez-Chapula et al.^22^ |
| *I_CaL_* | 1036.7 | 1.00 | Yasuda et al.^21^ |

Reduction of ionic currents (% of original value used in the computer model) due to the introduction of disopyramide. IC_50_ indicates the half-maximal inhibitory concentration; nH, Hill coefficient.

**Table 5. The effects of quinidine with a concentration of 4 μM on ionic currents.**

| Ionic currents | IC_50_(μM) | nH | References based |
| --- | --- | --- | --- |
| *I_Kr_* | 0.62±0.03 | 0.93±0.06 | McPate et al.^19,20^ |
| *I_Ks_* | 4.899 | 1.40 | Crumb et al.^23^ |
| *I_to_* | 3.487 | 1.30 | Crumb et al.^23^ |
| *I_Na_* | 14.6 | 1.22 | Kramer et al.^24^ |
| *I_CaL_* | 14.9±1.5 | 1.10±0.10 | Zhang et al.^25^ |

Reduction of ionic currents (% of original value used in the computer model) due to the introduction of quinidine. IC_50_ indicates the half-maximal inhibitory concentration; nH, Hill coefficient.

**Table 6. The effects of digoxin with a concentration of 1 nM on ionic currents, transporters and exchangers.**

| Ionic currents | IC_50_(nM) | nH | References based |
| --- | --- | --- | --- |
| *I_NaK_* | 120±20 | 1 | Pullen et al.^26^ |
| *I_Kr_* | 53.9±14.3 | 1 | Wang et al.^27^ |
| *RyR* | - | - | McGarry et al.^28^ |

**Table 7. Changes of ionic channels to simulate the effects of acetylcholine (ACH, 10 nM) and isoprenaline (ISO, 1.0 μM)**.

| Ionic currents | ACH (10 nM) | ISO (0.848 μM) | ISO (1.0 μM) |
| --- | --- | --- | --- |
| *I_f_* | Shift of *y_∞_* and *τ_y_* by -5 mV | Shift of *y_∞_* and *τ_y_* by 6.36 mV | Shift of *y_∞_* and *τ_y_* by 7.5 mV |
| *I_CaL_* | 3% reduction of maximal conductance | 19.50% increase of maximal conductance;  Shift *dL_∞_* and *τ_dL_* by -6.784 mV;  22.896% reduction of *K_dL_* | 23% increase of maximal conductance;  Shift *dL_∞_* and *τ_dL_* by -8 mV;  27% reduction of *K_dL_* |
| *I_Ks_* | - | 16.96% increase of maximal conductance;  Shift of *n_∞_* and *τ_n_* by -11.872 mV; | 20% increase of maximal conductance;  Shift of *n_∞_* and *τ_n_* by -14 mV; |
| *I_KACh_* | Activation | Inactivation | Inactivation |
| *I_NaK_* | - | 16.96% increase of maximal conductance | 20% increase of maximal conductance |
| *J_up_* | 7% reduction of maximal conductance | 21.2% increase of maximal conductance | 25% increase of maximal conductance |

The percentage of changes was based on normal sinoatrial node cell model. Here *y_∞_* indicates steady-state for gating variable *y* of *I_f_*; *τ_y_*, time constant for gating variable *y* of *I_f_*; *dL_∞_*, steady-state for voltage-dependent activation gating variable *dL* of *I_CaL_*; *τ_dL_*, time constant for voltage-dependent activation gating variable *dL* of *I_CaL_*; *K_dL_*, the slope factor for voltage-dependent activation gating variable *dL* of *I_CaL_*; *n_∞_*, steady-state for gating variable *n* of *I_Ks_*; *τ_n_*, time constant for gating variable *n* of *I_Ks_*.

**Table 8. Changes to our human atrial model to simulate atrial fibrillation^4^.**

| Ionic current | AF Versus Sinus Rhythm | Changes in parameters |
| --- | --- | --- |
| *I_Na_* | -10% peak density^29^ | 0.9**G_Na_* |
| *I_Ks_* | Increased 2-fold^30^ | 2.0**G_Ks_* |
| *I_Kur_* | -50% in the RA^30,31^ | 0.5**G_Kur_* |
| *I_K1_* | Upregulated +100%^31,32^ | 2.0**G_K1_* |
| *I_to_* | -80% in the RA^30,31^ | 0.2* *G_to_* |
| *I_CaL_* | Current density is reduced by 50%^31,33,34^ | 0.5* *G_CaL_* |
| *I_NCX_* | Upregulated in AF (+40%)^35,36^ | 1.4**G_NCX_* |
| *J_up_* | Reduced maximal pump rate^37,38^ | 3.0* *k_CaSR_*  1.25**V_leak_* |
| *J_rel_* | Increased sensitivity for luminal Ca^2+^ (2-fold)^37^ |  |
| *J_leak_* | Increased by 25%^37^ |  |

**Table 9. Action potential (AP) features of sinoatrial node cells with changes in ionic currents, transporters and exchangers.**

|  | MDP (mV) | Heart rate (beats/min) | (dV/dt)_max_ (mV/ms) | OS (mV) | DDR_100_ (mV/s) | (Cai)_min_(mM) | (Cai)_max_(mM) |
| --- | --- | --- | --- | --- | --- | --- | --- |
| *I_f_* (100~0%) | -58.9~-59.3 | 73.7~57.7 | 7.5~7.56 | 26.4~26.74 | 56.7~40.9 | 8.4E-5~6.9E-5 | 1.89E-4~1.72E-4 |
| *I_Na_*(100~0%) | -58.9~-59.1 | 73.7~64.6 | 7.5~7.47 | 26.4~26.38 | 56.7~50.35 | 8.4E-5~7.6E-5 | 1.89E-4~1.79E-4 |
| *I_CaT_*(100~5%) | -58.9~-64.0 | 73.7~33.3 | 7.5~7.87 | 26.4~26.73 | 56.7~23.85 | 8.4E-5~3.7E-5 | 1.89E-4~1.4E-4 |
| *I_CaL_*(100~48%) | -58.9~-56.37 | 73.7~46.6 | 7.5~2.92 | 26.4~10.30 | 56.7~50.10 | 8.4E-5~7.2E-5 | 1.89E-4~1.81E-4 |
| *I_Ks_*(100~0%) | -58.9~-59.0 | 73.7~73.8 | 7.5~7.51 | 26.4~26.74 | 56.7~58.12 | 8.4E-5~8.4E-5 | 1.89E-4~1.89E-4 |
| *I_Kr_*(100~0%) | -58.9~-37.1 | 73.7~117.8 | 7.5~2.69 | 26.4~14.86 | 56.7~92.75 | 8.4E-5~1.05E-4 | 1.89E-4~1.63E-4 |
| *I_to_*(100~0%) | -58.9~-58.85 | 73.7~86.3 | 7.5~7.7 | 26.4~28.69 | 56.7~61.25 | 8.4E-5~9.3E-5 | 1.89E-4~2E-4 |
| *I_Kur_*(100~0%) | -58.9~-59.64 | 73.7~68.2 | 7.5~7.55 | 26.4~30.32 | 56.7~20.55 | 8.4E-5~7.9E-5 | 1.89E-4~1.81E-4 |
| *I_NaK_*(100~0%) | -58.9~-58.07 | 73.7~109.8 | 7.5~7.86 | 26.4~30.30 | 56.7~68.2 | 8.4E-5~1.13E-4 | 1.89E-4~2.31E-4 |
| *I_NCX_*(100~25%) | -58.9~-57.43 | 73.7~52.44 | 7.5~2.89 | 26.4~10.24 | 56.7~55.0 | 8.4E-5~1.87E-4 | 1.89E-4~4.22E-4 |
| *J_rel_*(100~0%) | -58.9~-59.23 | 73.7~70.4 | 7.5~7.37 | 26.4~27.33 | 56.7~56.55 | 8.4E-5~7.8E-5 | 1.89E-4~1.68E-4 |
| *J_up_*(100~0%) | -58.9~-58.56 | 73.7~77.8 | 7.5~7.26 | 26.4~27.05 | 56.7~57.0 | 8.4E-5~9.9E-5 | 1.89E-4~2.09E-4 |

**Table 10. Action potential (AP) features of atrial cells with changes in ionic currents, transporters and exchangers.**

|  | MDP (mV) | APD_90_ (ms) | (dV/dt)_max_ (mV/ms) | OS (mV) | (Cai)_min_(mM) | (Cai)_max_(mM) |
| --- | --- | --- | --- | --- | --- | --- |
| *I_Na_*(100~90%) | -77.63~-77.63 | 233.64~233.92 | 164.51~150.19 | 24.26~23.46 | 1.45E-4~1.45E-4 | 4.98E-4~4.98E-4 |
| *I_CaL_*(100~48%) | -77.63~-79.74 | 233.64~208.56 | 164.51~221.11 | 24.26~27.22 | 1.45E-4~1.09E-4 | 4.98E-4~2.37E-4 |
| *I_Ks_*(100~200%) | -77.63~-78.37 | 233.64~195.4 | 164.51~183.55 | 24.26~25.61 | 1.45E-4~1.32E-4 | 4.98E-4~4.18E-4 |
| *I_K1_*(100~200%) | -77.63~-82.67 | 233.64~223.72 | 164.51~287.79 | 24.26~34.40 | 1.45E-4~1.35E-4 | 4.98E-4~4.54E-4 |
| *I_to_*(100~20%) | -77.63~-78.21 | 233.64~224.92 | 164.51~169.24 | 24.26~29.02 | 1.45E-4~1.42E-4 | 4.98E-4~4.78E-4 |
| *I_Kur_*(100~50%) | -77.63~-78.86 | 233.64~239.9 | 164.51~159.79 | 24.26~24.28 | 1.45E-4~1.48E-4 | 4.98E-4~5.07E-4 |
| *I_NCX_*(100~140%) | -77.63~-76.86 | 233.64~237.2 | 164.51~142.62 | 24.26~23.23 | 1.45E-4~1.13E-4 | 4.98E-4~3.7E-4 |
| *J_leak_*(100~125%) | -77.63~-77.64 | 233.64~231.7 | 164.51~153.53 | 24.26~23.58 | 1.45E-4~1.53E-4 | 4.98E-4~4.59E-4 |
| *k_CaSR_*(100~300%) | -77.63~-78.47 | 233.64~240.56 | 164.51~182.03 | 24.26~25.46 | 1.45E-4~1.33E-4 | 4.98E-4~6.03E-4 |

1. **Supplementary Figures**

**
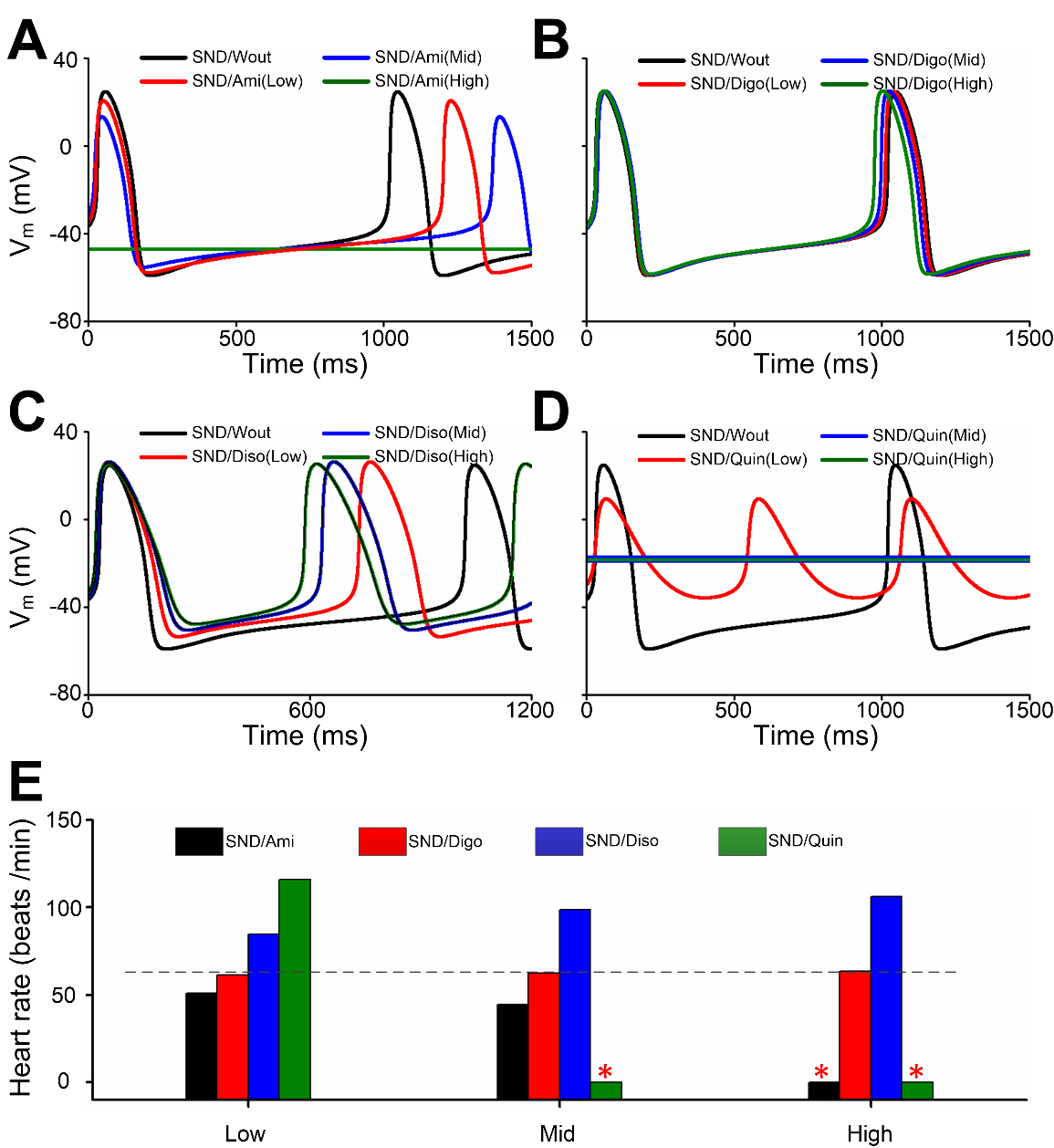
**

**Figure 1. The concentration-dependent effects of drugs on action potentials (Vm) and heart rates.**(**A**) Effects of amiodarone (SND/Ami) at the low (SND/Ami/Low), middle (SND/Ami/Mid), and high (SND/Ami/High) concentrations. (**B**) Effects of digoxin (SND/Digo) at different concentrations on action potentials. (**C-D**) Effects of disopyramide (SND/Diso) and quinidine (SND/Quin). (**E**) Effects of drugs on heart rate. Red stars indicate heart arrest, and the black dash line indicates the heart rate in the sinus node dysfunction (SND) condition without drugs.

**
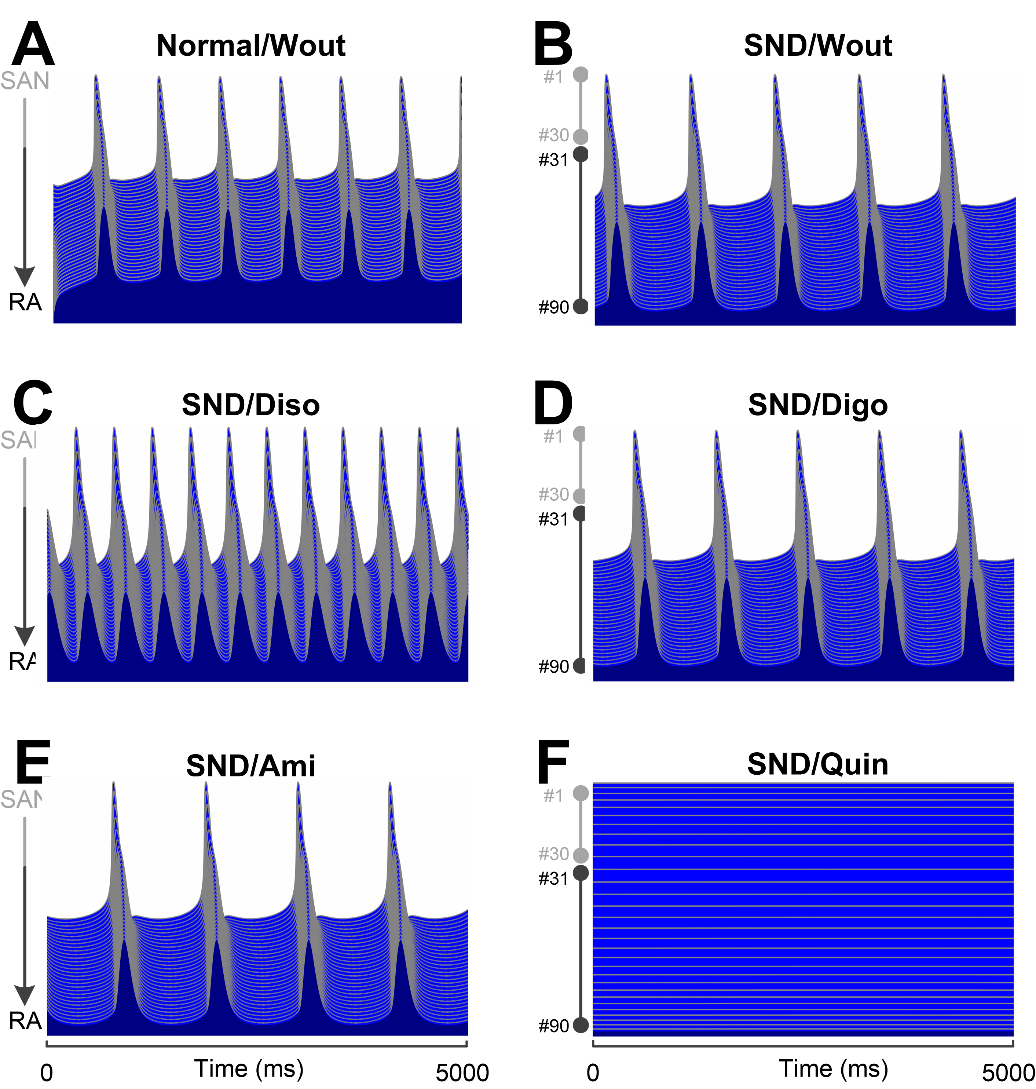
**

**Figure 2. One-dimensional simulations of electrical waves in drug-free settings versus in the presence of drugs.** For each set of panels, the one-dimensional strand contains 30 sinoatrial node (SAN, #1-#30) cells and 60 atrial (RA, #31-#90) cells. Electrical waves propagated from the SAN region to the RA region. Compared with the normal condition (**A**), (**B**) Heart beats decreased under the SND conditions;(**C**) An increase in heart beats in the presence of disopyramide; (**D**) No changes in heart beats in the presence of digoxin; (**E**) A reduction in heart rates in the presence of amiodarone; (**F**) SAN arrest in the presence of quinidine.

**
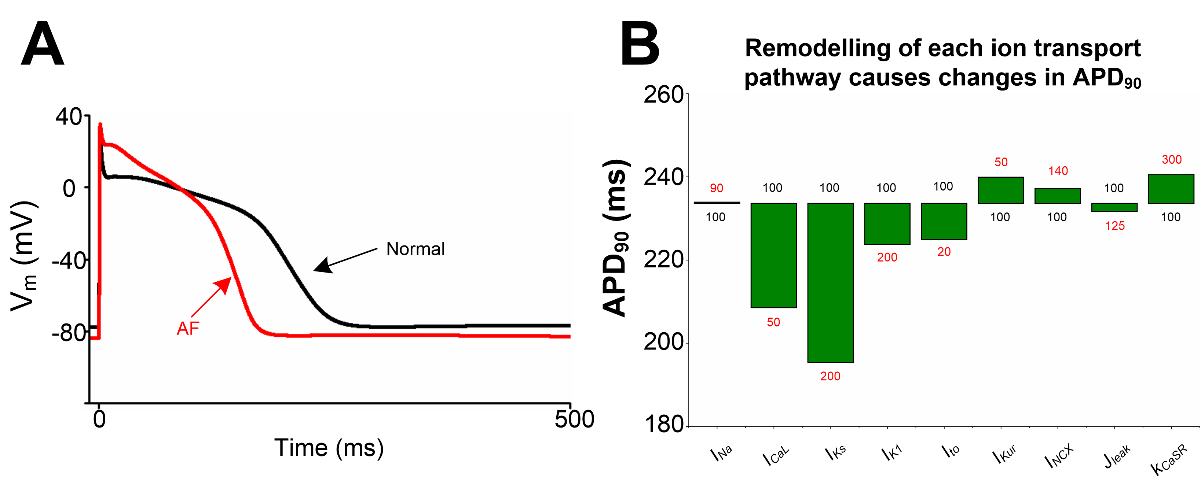
**

**Figure 3. Action potential (AP)s of atrial cells and changes in APD_90._ (A)** APs under normal and atrial fibrillation (AF) conditions. (**B**) Changes in APD_90_ when each ion current is remodelled from 100% (black number) to *x%* (red number) in atrial cells.

1. **References**

1 Fabbri, A., Fantini, M., Wilders, R. & Severi, S. Computational analysis of the human sinus node action potential: model development and effects of mutations. *The Journal of physiology* **595**, 2365-2396 (2017).

2 Yeh, Y.-H. *et al.* Funny current downregulation and sinus node dysfunction associated with atrial tachyarrhythmia: a molecular basis for tachycardia-bradycardia syndrome. *Circulation* **119**, 1576-1585 (2009).

3 Joung, B. *et al.* Mechanisms of sinoatrial node dysfunction in a canine model of pacing-induced atrial fibrillation. *Heart Rhythm* **7**, 88-95 (2010).

4 Grandi, E. *et al.* Human atrial action potential and Ca2+ model: sinus rhythm and chronic atrial fibrillation. *Circulation research* **109**, 1055-1066 (2011).

5 Bai, J., Gladding, P. A., Stiles, M. K., Fedorov, V. V. & Zhao, J. Ionic and cellular mechanisms underlying TBX5/PITX2 insufficiency-induced atrial fibrillation: Insights from mathematical models of human atrial cells. *Scientific reports* **8**, 15642 (2018).

6 Ten Tusscher, K. H. & Panfilov, A. V. Alternans and spiral breakup in a human ventricular tissue model. *American Journal of Physiology-Heart and Circulatory Physiology* **291**, H1088-H1100 (2006).

7 Garny, A. *et al.* Advanced computational model of central and peripheral rabbit sino-atrial node cells. *Biophysical Journal* **78**, 2674-2674 (2000).

8 Zhang, H. *et al.* Mathematical models of action potentials in the periphery and center of the rabbit sinoatrial node. *American Journal of Physiology-Heart and Circulatory Physiology* **279**, H397-H421 (2000).

9 Lalevée, N., Barrère‐lemaire, S., Gautier, P., Nargeot, J. & Richard, S. Effects of Amiodarone and Dronedarone on Voltage‐Dependent Sodium Current in Human Cardiomyocytes. *Journal of cardiovascular electrophysiology* **14**, 885-890 (2003).

10 Fan, X. *et al.* Novel electropharmacological activity of amiodarone on human HCN channels heterologously expressed in the Xenopus oocytes. *European journal of pharmacology* **669**, 15-23 (2011).

11 Heijman, J., Heusch, G. & Dobrev, D. Pleiotropic effects of antiarrhythmic agents: dronedarone in the treatment of atrial fibrillation. *Clinical Medicine Insights: Cardiology* **7**, CMC. S8445 (2013).

12 Yamashita, N. *et al.* Short-and long-term amiodarone treatments regulate Cav3. 2 low-voltage-activated T-type Ca2+ channel through distinct mechanisms. *Molecular pharmacology* **69**, 1684-1691 (2006).

13 Varró, A., Virág, L. & Papp, J. G. Comparison of the chronic and acute effects of amiodarone on the calcium and potassium currents in rabbit isolated cardiac myocytes. *British journal of pharmacology* **117**, 1181-1186 (1996).

14 Kamiya, K. *et al.* Short-and long-term effects of amiodarone on the two components of cardiac delayed rectifier K+ current. *Circulation* **103**, 1317-1324 (2001).

15 Kobayashi, S. *et al.* Inhibitory effect of bepridil on hKv1. 5 channel current: comparison with amiodarone and E-4031. *European journal of pharmacology* **430**, 149-157 (2001).

16 Watanabe, Y., Hara, Y., Tamagawa, M. & Nakaya, H. Inhibitory effect of amiodarone on the muscarinic acetylcholine receptor-operated potassium current in guinea pig atrial cells. *Journal of Pharmacology and Experimental Therapeutics* **279**, 617-624 (1996).

17 Watanabe, Y. & Kimura, J. Inhibitory effect of amiodarone on Na+/Ca2+ exchange current in guinea‐pig cardiac myocytes. *British journal of pharmacology* **131**, 80-84 (2000).

18 Gray, D. F. *et al.* Amiodarone inhibits the Na+-K+ pump in rabbit cardiac myocytes after acute and chronic treatment. *Journal of Pharmacology and Experimental Therapeutics* **284**, 75-82 (1998).

19 McPate, M. J., Duncan, R. S., Witchel, H. J. & Hancox, J. C. Disopyramide is an effective inhibitor of mutant HERG K+ channels involved in variant 1 short QT syndrome. *Journal of molecular and cellular cardiology* **41**, 563-566 (2006).

20 McPate, M., Duncan, R., Hancox, J. & Witchel, H. Pharmacology of the short QT syndrome N588K‐hERG K+ channel mutation: differential impact on selected class I and class III antiarrhythmic drugs. *British journal of pharmacology* **155**, 957-966 (2008).

21 Yasuda, C. *et al.* The human ether-a-go-go-related gene (hERG) current inhibition selectively prolongs action potential of midmyocardial cells to augment transmural dispersion. *J Physiol Pharmacol* **66**, 599-607 (2015).

22 Sanchez-Chapula, J. A. Mechanism of transient outward K+ channel block by disopyramide. *Journal of Pharmacology and Experimental Therapeutics* **290**, 515-523 (1999).

23 Crumb, W. J., Vicente, J., Johannesen, L. & Strauss, D. G. An evaluation of 30 clinical drugs against the comprehensive in vitro proarrhythmia assay (CiPA) proposed ion channel panel. *Journal of pharmacological and toxicological methods* **81**, 251-262 (2016).

24 Kramer, J. *et al.* MICE models: superior to the HERG model in predicting Torsade de Pointes. *Scientific reports* **3**, 2100 (2013).

25 Zhang, Y. & Hancox, J. Mode‐dependent inhibition by quinidine of Na+–Ca2+ exchanger current from guinea‐pig isolated ventricular myocytes. *Clinical and experimental pharmacology and physiology* **29**, 777-781 (2002).

26 Pullen, M. A., Brooks, D. P. & Edwards, R. M. Characterization of the neutralizing activity of digoxin-specific Fab toward ouabain-like steroids. *Journal of Pharmacology and Experimental Therapeutics* **310**, 319-325 (2004).

27 Wang, L., Wible, B. A., Wan, X. & Ficker, E. Cardiac glycosides as novel inhibitors of human ether-a-go-go-related gene channel trafficking. *Journal of Pharmacology and Experimental Therapeutics* **320**, 525-534 (2007).

28 McGarry, S. J. & Williams, A. J. Digoxin activates sarcoplasmic reticulum Ca2+‐release channels: a possible role in cardiac inotropy. *British journal of pharmacology* **108**, 1043-1050 (1993).

29 Sossalla, S. *et al.* Altered Na+ currents in atrial fibrillation: effects of ranolazine on arrhythmias and contractility in human atrial myocardium. *Journal of the American College of Cardiology* **55**, 2330-2342 (2010).

30 Caballero, R. *et al.* In humans, chronic atrial fibrillation decreases the transient outward current and ultrarapid component of the delayed rectifier current differentially on each atria and increases the slow component of the delayed rectifier current in both. *Journal of the American College of Cardiology* **55**, 2346-2354 (2010).

31 Dobrev, D. & Ravens, U. Remodeling of cardiomyocyte ion channels in human atrial fibrillation. *Basic research in cardiology* **98**, 137-148 (2003).

32 Li, G. & Nattel, S. Properties of human atrial ICa at physiological temperatures and relevance to action potential. *American Journal of Physiology-Heart and Circulatory Physiology* **272**, H227-H235 (1997).

33 Christ, T. *et al.* L-type Ca2+ current downregulation in chronic human atrial fibrillation is associated with increased activity of protein phosphatases. *Circulation* **110**, 2651-2657 (2004).

34 Van Wagoner, D. R. *et al.* Atrial L-type Ca2+ currents and human atrial fibrillation. *Circulation research* **85**, 428-436 (1999).

35 Schotten, U. *et al.* Atrial fibrillation-induced atrial contractile dysfunction: a tachycardiomyopathy of a different sort. *Cardiovascular research* **53**, 192-201 (2002).

36 Wang, J. *et al.* Regional expression of sodium pump subunits isoforms and Na+-Ca++ exchanger in the human heart. *The Journal of clinical investigation* **98**, 1650-1658 (1996).

37 Voigt, N. *et al.* Cellular and molecular mechanisms of atrial arrhythmogenesis in patients with paroxysmal atrial fibrillation. *Circulation* **129**, 145-156 (2014).

38 Bokník, P. *et al.* Regional expression of phospholamban in the human heart. *Cardiovascular research* **43**, 67-76 (1999).
